# High prevalence of long-term olfactory dysfunction confirmed by olfactory testing after a community COVID-19 outbreak

**DOI:** 10.1007/s00106-021-01129-7

**Published:** 2021-12-23

**Authors:** Hilmar Gudziol, Timo Kirschstein, Mathias W. Pletz, Sebastian Weis, Orlando Guntinas-Lichius, Thomas Bitter, the CoNAN study group, Thomas Hotz, Petra Enders, Renate Koch, Steffen Mai, Matthias Ullrich, Cora Richert, Cornelius Eibner, Bettina Meinung, Kay Stötzer, Julia Köhler, Hans Cipowicz, Christine Pinkwart, Michael Bauer, Petra Dickmann, Annika Licht, Juliane Scholz, Wibke Wetzker, Anita Hartung, Daniel Weiss, Lara Thieme, Gabi Hanf, Clara Schnizer, Jasmin Müller, Jennifer Kosenkow, Franziska Röstel, Nico Andreas, Raphaela Marquardt, Stefanie Deinhardt-Emmer, Sebastian Kuhn

**Affiliations:** 1 Department of Otorhinolaryngology, Jena University Hospital, Friedrich Schiller University, Am Klinikum 1, 07740 Jena, Germany; 2 Institute for Infectious Diseases and Infection Control, Jena University Hospital, Friedrich Schiller University, Jena, Germany; 3 Center for Sepsis Control and Care (CSCC), Jena University Hospital, Friedrich Schiller University, Jena, Germany; 4 Department of Anesthesiology and Intensive Care, Jena University Hospital, Friedrich Schiller University, Jena, Germany

**Keywords:** Coronavirus, Outbreak, Smell, Olfaction, Long-term sequelae, Coronavirus, Ausbruch, Geruch, Schmecken, Langzeitfolgen

## Abstract

**Purpose:**

The prevalence of long-term olfactory and gustatory dysfunction in participants suffering from sudden chemosensory loss due to coronavirus disease 2019 (COVID-19) is unknown. Furthermore, evaluations of the reliability of participants’ self-reporting of olfactory function (SOF) and gustatory function (SGF) using extended objective psychophysical testing are missing.

**Methods:**

In this population-based cohort study in a PCR-tested community in Thuringia, Germany, olfactory function was extensively examined 4 months after a COVID-19 outbreak using the “Sniffin Sticks” test battery to determine the TDI_a_ score, i.e., the sum of results obtained for threshold, discrimination, and identification scores averaged for both nasal sides. Gustatory function was assessed using the three-drop test resulting in the gustatory composite score (CS_g_). The data were compared with SOF and SGF.

**Results:**

Of 43 adult convalescents (median age: 68 years; 58% female) after SARS-CoV‑2 infection, 18 participants (42%) had olfactory complaints due to SOF, one participant (2%) complained of taste disturbance due to SGF. The TDI_a_ was 22.0 ± 5.9. Normosmia, hyposmia, and anosmia were seen in 17, 18, and eight participants, respectively. TDI_a_ correlated with SOF (*r*_s_ = −0.434, *p* = 0.004); CS_g_ was 23.5 ± 2.7. Normogeusia and hypogeusia were objectified in 39 and four participants, respectively. The prevalence of long-term olfactory dysfunction and gustatory dysfunction in the study group was 60.5 and 9.3%, respectively.

**Conclusion:**

The SOF was reliable, especially for participants who felt a sudden chemosensory dysfunction during the outbreak. At 4 months after SARS-CoV‑2 infection, a high proportion of participants were dysosmic, whereas nearly all of them had normal taste function.

## Abstract (de)

**Ziel:**

Die Prävalenz einer langfristigen olfaktorischen und gustatorischen Dysfunktion bei Patienten, die initial aufgrund der Coronavirus-Krankheit 2019 (COVID-19) einen plötzlichen chemosensorischen Verlust erlitten haben, ist unbekannt. Darüber hinaus fehlen Auswertungen zur Zuverlässigkeit der Selbstauskunft der Patienten zur Geruchsfunktion (SOF) und zur Schmeckfunktion (SGF) mittels erweiterter psychophysischer Tests.

**Methoden:**

In dieser populationsbasierten Kohortenstudie in einer PCR-getesteten Gemeinde in Thüringen wurde die Riechfunktion 4 Monate nach einem COVID-19-Ausbruch standardisiert mit „Sniffin-Sticks-Testbatterien“ zur Bestimmung des TDI_a_-Scores getestet, d. h. die Summe der Ergebnisse, die für Schwellenwert‑, Diskriminierungs- und Identifizierungstest erhalten wurden, gemittelt für beide Nasenseiten. Die gustatorische Funktion wurde mit dem 3‑Tropfen-Test getestet, der einen Gustatory Composite Score (CS_g_) ergab. Die Daten wurden mit SOF und SGF verglichen.

**Ergebnisse:**

Von 43 erwachsenen Rekonvaleszenten (mittleres Alter: 68 Jahre; 58 % weiblich) mit SARS-CoV-2-Infektion berichteten 18 Teilnehmer (42 %) über eine anhaltende Geruchsminderung im SOF, ein Teilnehmer (2 %) klagte über eine Schmeckstörung im SGF. TDI_a_ betrug 22,0 ± 5,9. Normosmie, Hyposmie und Anosmie wurden bei 17, 18 bzw. 8 Patienten beobachtet. TDI_a_ korrelierte mit SOF (rs = −0,434; *p* = 0,004). CS_g_ betrug 23,5 ± 2,7. Normogeusie und Hypogeusie wurden bei 39 bzw. 4 Patienten objektiviert. Die Prävalenz langfristiger Riech- und Geschmacksstörungen in der Studiengruppe betrug 60,5 bzw. 9,3 %.

**Schlussfolgerungen:**

Der SOF erwies sich als besonders zuverlässig geeignet für Patienten, die während des Ausbruchs eine plötzliche chemosensorische Dysfunktion bemerkt haben. Ein hoher Anteil der Teilnehmer war 4 Monate nach der SARS-CoV-2-Infektion immer noch dysosmisch, während fast alle eine normale Schmeckfunktion aufwiesen.

Sudden acquired olfactory loss is uncommon. Postinfectious olfactory loss is the most frequent reason. Since infection-associated olfactory loss is usually completely reversible, patients typically visit a physician only months or even years later and only in the exceptional case of long-term complaints [1]. However, this appears to be different for COVID-19 convalescent participants. In these patients the sudden loss of smell and loss of taste even without further symptoms is reminiscent of a respiratory tract infection and has been reported more frequently than for other viral infections [2, 3]. Thus, it is considered a typical symptom of COVID-19 disease [4, 5]. There are only few studies addressing this complication with a potential high impact on the quality of life. There is no information on the long-term prognosis of smell and taste impairment after SARS-CoV‑2 infection.

The question that arises first is whether the subjectively experienced and reported sensation of an acute smell loss or taste loss is sufficient to justify the measure of a laboratory SARS-CoV‑2 test and to initiate quarantine and even therapy adapted to the severity of the disease. Second, it should be investigated whether participants’ self-ratings are reliable also for follow-up investigations to assess the irreversibility or reversibility of the complaints. Validated psychophysical tests are the current gold standard in testing for smell and taste disorders. Given the complexity of these procedures, it is difficult to realize such psychophysical tests for all participants with suspected SARS-CoV‑2 infection. Furthermore, such testing exposes the medical staff to direct risk of infection. It would be attractive to have tests available that the participant can use himself/herself [6, 7]. These tests are often quite expensive and are not available ad hoc everywhere [8, 9]. Additionally, there is a lack of data demonstrating the reliability of participants’ self-ratings. On the one hand, one quarter of participants with anosmia seem to be unaware of their anosmia and one -third of participants indicating olfactory loss have an inconspicuous test result when using validated psychophysical tests [10].

Therefore, this study aimed to investigate the prevalence of smell and taste dysfunction in individuals from the CoNAN study (Covid-19 Outbreak in Neustadt-am-Rennsteig [11]) who recovered from SARS-CoV‑2 infection occurring 4 months earlier. Furthermore, we aimed to assess the reliability of participants’ self-rating of olfactory function (SOF) and of gustatory function (SGF) s after SARS-CoV‑2 infection in comparison with validated psychophysical tests. If SOF and SGF are proven reliable, further chemosensory testing may be postponed and much simpler SOF and SGF could be used for further diagnosis and treatment decision-making.

## Methods

### Study design and participants

**Fig. 1:**
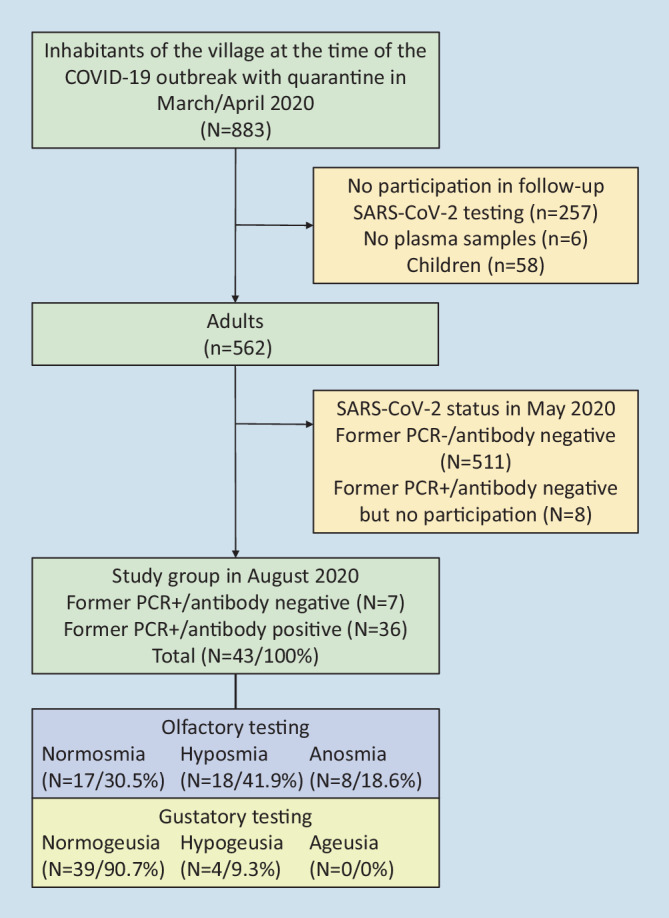
Flow chart of the study

The examinations were carried out by four trained examiners in well-ventilated rooms from August 17, 2020 to August 21, 2020. The local ethics committee approved the study (registration number: 2020-1770-BO). Participants were informed about the test procedure and written informed consent was obtained. All participants were recruited from the CoNAN study cohort (Covid-19 Outbreak in Neustadt-am-Rennsteig), a longitudinal cohort study analyzing the course of SARS-CoV‑2 infections in Neustadt-am-Rennsteig, a village in Thuringia, Germany [11]. The flowchart of the study is presented in Fig. 1. The local public health authorities had declared a 14-day quarantine (March 22, 2020 to April 5, 2020) for the entire village of 883 inhabitants with ultimately 49 confirmed SARS-CoV‑2 infections in the adult population (positive SARS-CoV‑2 PCR rate: 8.7% of 562 adults tested). The inclusion criteria for the pre-selection for this chemosensory study were proof of a previous SARS-CoV‑2 infection by a laboratory, either by positive SARS-CoV‑2 polymerase chain reaction (PCR) tests or the detection of SARS-CoV‑2 immunoglobulin G (IgG) antibodies using six different tests (details in [11]). Individuals with a previously confirmed SARS-CoV‑2 infection were contacted by telephone and asked for study participation. Finally, 43 persons with confirmed and recovered SARS-CoV‑2 infections were included. Of these, 36 participants had a positive antibody test result in May 2020. Seven participants reported positive SARS-CoV‑2 PCR test results during March–April. All 43 adults had a negative SARS-CoV‑2 PCR test result in May 2020. At the time of the examinations in August 2020, none of the participants reported symptoms compatible with an acute respiratory tract infection. Nineteen participants (two smokers) stated that they had experienced a chemosensory disturbance in connection with their corona infection. In this subgroup, 18 participants tested positive for SARS-CoV‑2 IgG antibodies (15 times in six tests, once in three tests, and two times in one test). Overall, 24 formerly infected participants (four smokers) did not complain of any chemosensory disturbances during the phase of acute infection. In this group, 18 participants tested positive for SARS-CoV‑2 IgG antibodies (five times positive in six tests, twice positive in two tests, and 11 positive in one test). The difference in the SARS-CoV‑2 IgG antibodies (SAB) distribution was significant between the two groups (Mann–Whitney *U*-test, *p* < 0.001). In participants with chemosensory complaints, SAB was more frequent than in participants without chemosensory complaints.

### Olfactory testing

The ability to smell in both sides of the nose was tested using the standardized and validated psychophysical extended Sniffin’ Sticks test assay [12]. This smell test assesses the three main components of olfactory function, namely, (a) the perception of odorants at low concentrations (odor threshold), (b) the distinction of different smells (odor discrimination), and (c) the ability to name or associate an odorant (odor identification). The detailed procedure has been described previously [13, 14]. The sum of odor threshold, discrimination, and identification scores (TDI score) for each side of the nose was determined. An average TDI score for each individual was then calculated from both nasal sides (TDI_a_). Normative TDI_a_ values were defined in relation to gender and age [15]. The results were classified as follows: normal olfactory function was defined as ≥10th percentile of the gender- and age-related TDI_a_; reduced olfactory function was defined as <10th percentile of the gender- and age-related TDI_a_; and functional anosmia was defined as ≤16 TDI_a_.

### Gustatory testing

Using the three-drop gustatory test [16], the recognition threshold of the four basic taste qualities sweet, sour, salty, and bitter assessed in six dilution steps (sucrose [% v/v]: 0.75, 1.5, 3, 10, 40, cold saturated; citric acid [% v/v]: 0.25, 0.5, 1, 5, 10, 15; sodium chloride [% v/v]: 0.6, 1.2, 2.5, 7.5, 15, cold saturated; quinine hydrochloride [% v/v]: 0.005, 0.01, 0.02, 0.05, 0.1, 1) was analyzed and scored. Distilled water was used as solvent. Taste solutions were prepared freshly and retained in brown glass bottles with pipettes. One drop of each taste quality in ascending concentrations was given on the protruded tongue to be tasted and swallowed until the test person recognized it correctly. The order of sweet, sour, and salty was random, and bitter was always tested in the end. Between trials, participants were allowed to drink a sip of tap water. The correctly recognized weakest concentration scored seven points and the highest concentration two points. If the highest concentration was not correctly recognized, the participants received one point. The sum of each basic gustatory score was a gustatory composite score (CS_g_). The results were classified as follows: normogeusia (normal taste) meant a CS_g_ ≥20 points, hypogeusia (impaired taste) <20 points ≥8 points, and ageusia (no taste) <8 points.

### Participants’ self-rating of olfactory function (SOF) and gustatory function (SGF)

The participants were interviewed about their general medical history, especially regarding their chemical senses. Concomitant common cold symptoms (CCCS) like fever, headache, rhinitis (running nose: four times; blocked nose: three times), sore throat, cough, sweats and chills, were summed up as positive CCCS and missing CCCS as negative. In interviewing the participants, it was important to prevent confusing taste and flavor. Such confusion arises often, because the flavor of a meal or a beverage is the result of retronasal smelling during swallowing of some food or drinks. Taste was explained to the participants as the ability to perceive sweet, sour, salty, or bitter. By intense questioning and clarification, the symptom complaints could be classified into olfactory or gustatory disturbances. Patients’ self-rating of olfactory function (SOF) and gustatory function (SGF) was categorized as follows: 1 = very good, 2 = good, 3 = moderate, or 4 = poor. All patients with chemosensory complaints were asked additionally about qualitative disorders like parosmia or parageusia during the presence of an odor or a taste. Finally, they were asked about phantosmia or phantogeusia, describing the subjective sensation of an odor or taste despite its absence.

### Statistical methods

The software IBM SPSS Statistics, version 24.0 (IBM Corp., Armonk, NY, USA) was used for statistical evaluation. For the TDI_a_ score, CS_g_ score, and age of the patients, the means and standard deviations (SD) of the study population were calculated. Nonparametric tests (Mann–Whitney *U*-test) were performed to analyze differences between independent subgroups of patients. Non-parametric tests were chosen because the data were not normally distributed and some measures were categorical. Spearman’s correlation coefficient was calculated to assess correlations between parameters. The significance level was set at *p* = 0.05.

## Results

The study group consisted of 43 adult patients (female: 58.1%; mean age: 62 ± 14.7). Of these patients, 19 self-reported a chemosensory impairment during the acute SARS-CoV‑2 infection and 24 did not self-report a chemosensory impairment during the community COVID-19 outbreak.

### Olfactory function 4 months after acute SARS-CoV-2 infection

An overview of the results of olfactory testing approximately 4 months after infection is given in Table 1. The TDI_a_ score averaged 22.0 ± 5.85 (range: 9.5–34.3). The age- and gender-related olfactory test result was 17 times normosmia, 18 times hyposmia, and 8 times anosmia. Related to the entire study group of former COVID-19-positive patients, the prevalence of long-term olfactory dysfunction was 60.5%.

**Table 1:** Results of olfactory psychophysical function tests 4 months after SARS-CoV‑2 infection

Parameter	All participants *N* = 43	Normal olfactory function *N* = 17	Reduced olfactory function *N* = 18	Anosmia *N* =8
*Age, years*				
Mean ± SD	62 ± 14.7	66.0 ± 15.7	55.9 ± 11.5	68.1 ± 15.2
*Gender, n*				
Female	25	9	11	5
Male	18	8	7	3
*CCCS, n*	20	8	9	3
*TDI*_*a*_* score*				
Mean ± SD	22.0 ± 5.9	26.2 ± 3.8	22.0 ± 3.7	13.0 ± 2.2
Range	9.5–34.3	17.0–34.3	16.25–28.0	9.5–15.8
*SOF, n*				
Very good	2	2	0	0
Good	26	13	8	5
Moderate	11	2	8	1
Poor	4	0	2	2
*CS*_*g*_* score*				
Mean ± SD	23.5 ± 2.7	23.7 ± 1.9	23.6 ± 3.1	22.9 ± 3.4
Range	15–28	19–27	15–27	19–28
*SGF*				
Very good	2	2	0	0
Good	26	13	9	4
Moderate	15	2	9	4
Poor	0	0	0	0

*TDI*_*a*_ average sum of odor thresholds, discrimination, and identification score for both sides of the nose, *CS*_*g*_ gustatory composite score, *SD* standard deviation, *SOF* participants’ self-rating of olfactory function, *SGF* participants’ self-rating of gustatory function, *CCCS* concomitant common cold symptoms

**Fig. 2:**
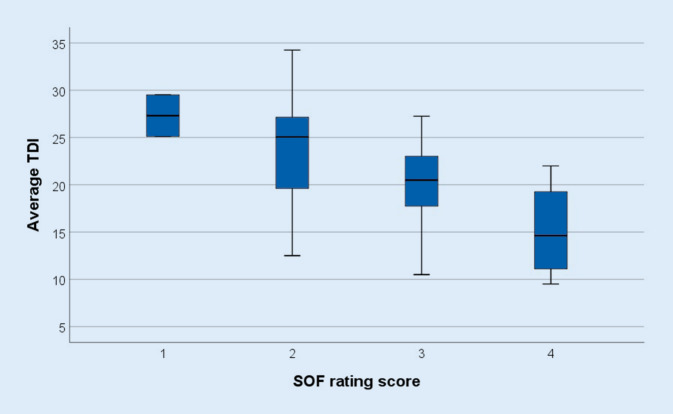
Boxplots showing the relation between participants’ self-rating of olfactory function (*SOF*) graded from 1 = “very good” to 4 = “poor” and olfactory testing (*TDI*_*a*_) 4 months after acute SARS-CoV‑2 infection

Three patients complained of a parosmia. SOF demonstrated “very good” two times, “good” 26 times, “moderate” 15 times, and “poor” zero times. In the whole group there was a moderate-to-strong negative correlation between the TDI_a_ score and SOF (Fig. 2; Spearman *r*_s_ = −0.434, *p* = 0.004). In patients reporting chemosensory complaints (*n* = 19) there was a strong negative correlation between TDI_a_ and SOF (Spearman *r*_s_ = −0.655, *p* = 0.002). In patients with CCCS (*n* = 20) there was a strong negative correlation between the TDI_a_ score and SOF (Spearman *r*_s_ = −0.688, *p* = 0.001). In only seven patients with rhinitis symptoms (*n* = 7) was there a significant negative correlation between TDI_a_ score and SOF (Pearson *r* = −0.775, *p* = 0.041). There was no difference in TDI_a_ score between patients with and without rhinitis (Mann–Whitney *U*-test, *p* = 0.211). In patients with positive SARS-CoV‑2 antibodies (*n* = 36) there was a strong negative correlation between TDI_a_ score and SOF (Spearman r_s_ = −0.484, *p* = 0.003).

A comparison of the patients reporting chemosensory dysfunction during the COVID-19 outbreak with the patients not reporting chemosensory dysfunction is presented in Table 2. These two subgroups were not different except for SOF assessed 4 months later: SOF results indicated better olfactory function in the subgroup of patients who did not complain of chemosensory dysfunction during the outbreak (*p* = 0.010). Interestingly, the rates for normal and disturbed olfactory function were not different between these two subgroups (*p* = 0.958). Three anosmic patients with chemosensory complaints had probably already felt a high-grade of reduced olfactory dysfunction before the corona infection, in which the residual olfactory ability worsened during the infection. A 71-year-old participant with chronic cardiovascular disease had a temporary anosmia. In another female participant of the same age, olfactory dysfunction was permanent. The first anosmic female participant described her SOF as good after the self-reported chemosensory impairments completely disappeared. The second female participant described it as poor because her decreased residual olfactory ability did not completely improve. A third 32-year-old woman with anosmia reported a reduced olfactory ability since childhood. She reported a residual olfactory ability that worsened during the corona infection. She felt that this had not yet completely improved, so that she rated it continuously as poor. Four of five patients (age range: 68–81 years) without chemosensory complaints but with measured functional anosmia rated their SOF as good and one as moderate, but nobody as poor. These five anosmic patients complained of other chronic illnesses: four had cardiovascular diseases, one person suffered from gout. Presumably, the olfactory ability of these five patients had been lost unnoticed gradually long before the SARS-CoV‑2 infection.

**Table 2:** Comparison of infected participants self-reporting chemosensory impairment versus participants without self-reported chemosensory during a former COVID-19 outbreak 4 months earlier

Parameter	Participants with self-reported chemosensory complaints during the outbreak *N* = 19	Participants without self-reported chemosensory complaints during the outbreak *N* = 24	*p*
*Age, years*			
Mean ± SD	60.7 ± 14.8	63.3 ± 14.8	0.441
*Gender, n*			
Female	14	11	0.069
Male	5	13	
*CCCS, n*	13	7	**0.011**
*TDI*_*a*_* score*			
Mean ± SD	21.9 ± 6.4	21.8 5.5	0.912
Range	9.5–34.3	10.5–29.5	
*Age- and gender-related olfactory function, n*			
Normal	6	10	0.958
Reduced	10	9	
Anosmia	3	5	
*SOF, n*			
Very good	0	2	**0.010**
Good	9	17	
Moderate	6	5	
Poor	4	0	
*CS*_*g*_* score*			
Mean ± SD	24.4 ± 2.1	22.5 ± 3.1	0.054
Range	19–28	15–27	
*Gustatory function, n*			
Normogeusia	19	20	0.065
Hypogeusia	0	4	
Ageusia	0	0	
*SGF, n*			
Very good	0	2	0.569
Good	12	14	
Moderate	7	8	
Poor	0	0	
*Parosmia, n*	3	0	NA
*Parageusia, n*	1	0	NA

*TDI*_*a*_ average sum of odor thresholds, discrimination, and identification score for both sides of the nose, *CS*_*g*_ gustatory composite score, *SD* standard deviation, *SOF* participants’ self-rating of olfactory function, *SGF* participants’ self-rating of gustatory function, *CCCS* concomitant common cold symptoms, *NA* not applicable

### Gustatory function 4 months after acute SARS-CoV-2 infection

Details on gustatory testing are displayed in Table 1. Gustatory examination showed a CS_g_ of 23.5 ± 2.7 (range: 15.0–28.0). Four patients had objective hypogeusia and 39 patients had objective normogeusia. This can be translated into a hypogeusia prevalence of 9.3% for the study population consisting of former COVID-19 patients.

Self-assessment of the gustatory function (SGF) resulted in the ratings “very good” two times, “good” 26 times, “moderate” 15 times, and “poor” zero times. There was no correlation between CS_g_ and SGF (Spearman *r*_s_ = −0.025, *p* = 0.874). None of the four patients with hypogeusia reported a chemosensory disturbance. All patients could correctly identify sweet, sour, and salty in different test concentrations. Only bitter was not correctly recognized by two patients (one with normogeusia and one with hypogeusia). The two patients, who did not recognize quinine hydrochloride as bitter, even in the strongest concentration, indicated no history of ageusia for bitter. These were very likely patients with pre-existing specific ageusia for quinine hydrochloride as described in the literature [16]. Persistent parageusia was perceived by one woman with normogeusia. She reported that everything tasted too salty. The same 72-year-old women reported transient ageusia for sweet and sour 4 months earlier, during her SARS-CoV‑2 infection.

## Discussion

At 4 months after a community COVID-19 outbreak, only 39.5% of the convalescents had a normal olfactory function. By contrast, 41.9% suffered from hyposmia and 18.6% even from anosmia. A limitation of the present study is that olfactory function before and during the outbreak is unknown. There is now strong evidence that many persons infected with SARS-CoV‑2 develop a loss of smell and taste function [17]. A problem seems to be that estimations of the prevalence of COVID-19-related smell and taste dysfunction are highly variable, because most studies relied on patients’ self-reporting of olfactory function (SOF) and gustatory function (SGF). Such self-report surveys are susceptible to confounding, e.g., by recall bias, sampling issues, and a lack of subject awareness [17]. For many persons recognizing less-than-total smell or taste is difficult to describe [18, 19]. This might be the reason why the reported prevalence rates for smell and/or taste dysfunction in SARS-CoV‑2 positive cases ranges from 5% to 85% [17]. In a recent meta-analysis, the estimated random prevalence of acute olfactory dysfunction was 43.0%, and that of taste dysfunction was 44.6% [20].

The problem of self-reporting applies also regarding estimates of recovery of function. Therefore, it is very important to focus on studies using well-validated and sensitive psychophysical tests to evaluate the prevalence and reversibility of olfactory and taste dysfunctions. In a study using the validated University of Pennsylvania Smell Identification Test (UPSIT) administered to 100 SARS-CoV-2-positive cases, 96% showed a measurable smell dysfunction. Out of these patients, 18% were anosmic in the hospital near the end of the acute phase of the disease [21]. Five weeks later, 63% of the retested patients had normal olfactory function. However, the mean UPSIT score at that time continued to remain below inconspicuous thresholds [21]. Another research group confirmed that olfactory dysfunction, using the short version of the Questionnaire of Olfactory Disorders-Negative Statements (sQOD-NS), persisted in 56% of cases 14 days after general symptom resolution [2]. Similarly, Vaira et al. used the Connecticut Chemosensory Clinical Research Center (CCCRC) orthonasal olfaction test and a screening taste solution test. They reported a persistence of alterations in 34% of cases during follow-up of an average of 21.7 days [22]. Using self-administered olfactory and gustatory psychophysical tests, Vaira et al. revealed that 60 days after onset, 7.2% of the patients still had anosmia and 4.2% a taste disorder [22]. We are aware of only a few other studies reporting follow-up data up to 4 months after onset or longer and all of them are hospital-based and not population-based. An 8‑month follow-up survey of 128 Israeli patients was published recently; 48% and 38.5% of patients reported a persistent smell and taste dysfunction, respectively [23]. The 9‑month follow-up data from Geneva, also only based on interviews, revealed a persistently experienced taste or smell loss in 16.8% of the patients [24]. Finally, using the Sniffin’ Sticks test in a group of 97 patients from Strasbourg, complete recovery of an initial smell loss was seen in 96.1% [25]. These data make clear how important it is to use psychophysical evaluations instead of nonvalidated questionnaires [26].

The reason for long-term effects of the SARS-CoV‑2 infection on olfactory function is unknown. Virus mutations may cause differing infectivity, while at the host level, genetic, ethnicity-specific variants of the virus-binding entry proteins may facilitate virus entry and a variable degree of destruction of the olfactory epithelium [20].

A strength of the present study was the focus on an enclosed and well-characterized community that was exposed to a COVID-19 outbreak [11]. Although the absolute number of patients is low, the population-based approach in contrast to larger hospital-based studies enables the direct deduction of healthcare-relevant data. Our approach made it possible to estimate the prevalence of long-term olfactory dysfunction within this community. It was 4.6% and 60.5%, respectively, related to all adult inhabitants of the community and to the subgroup of COVID-19 convalescents. We have probably overestimated the COVID-19-related effect, as one would expect a prevalence of olfactory dysfunction of approximately 19–26% in a normal population with the same age range [27]. If further studies confirm the present results, we have an expected worldwide surge of patients with persistent olfactory dysfunctions with a significant negative impact on quality of the life. Regarding the current literature, we cannot offer any causal olfactory treatment [28]. Our study was limited to adults and does not allow any statements to be made for children. Furthermore, most of the patients included in this study had a mild COVID-19 disease course. Hence, the results may be not representative of patients with severe COVID-19.

Our results confirm that the pathogenesis of taste disorders in COVID-19 patients is probably largely smell-independent [29]. Based on a validated test setting, it can be clearly confirmed that gustatory dysfunction is reversible in many cases or that a gustatory dysfunction never appeared. SGF is difficult for many patients, as many persons confuse the perception of flavored beverages and food during deglutition with tasting. The physiological term “taste” only describes the perception of the basic gustatory qualities. The fine taste of a food or drink is perceived retronasally via the olfactory sense. In fact, many experienced taste disorders prove to be olfactory dysfunctions.

The question remains whether SOF and SGF are reliable in the COVID-19 setting, or whether psychophysical testing is mandatory to evaluate smell and taste function. Recently, it was reported that a significant proportion of COVID-19 patients reporting an olfactory dysfunction do not have olfactory dysfunction based on objective testing [26]. The 16-identification Sniffin’ Sticks test used may be the cause of this. It was developed as a screening test and categorizes the individual olfactory function in a gross manner. Conversely, it is well known that many persons are not aware of their olfactory dysfunction [30]. This scenario also occurred in the present study. Nevertheless, there was a moderate-to-strong correlation between TDI_a_ score and SOF for the whole study population and a strong correlation when considering only patients with subjectively experienced chemosensory complaints. The reason seems to be that a sudden onset of an olfactory dysfunction that arose during acute SARS-CoV‑2 infection is perceived by an informed community with much higher attention than gradually developing olfactory disorders [31–33]. Overall, the present study showed that SOF and SGF seem to be sufficient for a rough average estimate of smell and taste function, but psychophysical testing cannot be omitted if a precise and individual time course of the dysfunction and its severity has to be monitored.

## Practical conclusion

- Olfactory dysfunction is a highly prevalent problem 4 months after acute SARS-CoV‑2 infection.
- A sudden chemosensory disorder is likely to be a strong hallmark of SARS-CoV‑2 infection during the pandemic.
- Self-reporting of olfactory function seems to be suitable for qualifying sudden olfactory loss in infected persons.
- Even 4 months after the onset of symptoms, there was a strong correlation between olfactory self-assessment and validated testing.

## Declarations

All procedures performed in studies involving human patients were in accordance with the ethical standards of the institutional and/or national research committee and with the 1964 Helsinki declaration and its later amendments or comparable ethical standards.

## References

[1] Cavazzana A, Larsson M, Munch M, Hahner A, Hummel T (2018). Postinfectious olfactory loss: a retrospective study on 791 participants. Laryngoscope.

[2] Lechien JR, Chiesa-Estomba CM, De Siati DR (2020). Olfactory and gustatory dysfunctions as a clinical presentation of mild-to-moderate forms of the coronavirus disease (COVID-19): a multicenter European study. Eur Arch Otorhinolaryngol.

[3] Borsetto D, Hopkins C, Philips V (2020). Self-reported alteration of sense of smell or taste in participants with COVID-19: a systematic review and meta-analysis on 3563 participants. Rhinology.

[4] Menni C, Valdes AM, Freidin MB (2020). Real-time tracking of self-reported symptoms to predict potential COVID-19. Nat Med.

[5] Joffily L, Ungierowicz A, David AG (2020). The close relationship between sudden loss of smell and COVID-19. Braz J Otorhinolaryngol.

[6] Besser G, Liu DT, Renner B, Mueller CA (2020). Self-administered testing of odor threshold and discrimination using sniffin’ sticks—reviving the “odor-curves-on-paper” method. Chemosens Percept.

[7] Mueller C, Kallert S, Renner B (2003). Quantitative assessment of gustatory function in a clinical context using impregnated “taste strips”. Rhinology.

[8] Forster G, Damm M, Gudziol H (2004). Olfactory dysfunction. Epidemiology, pathophsiological classification, diagnosis and therapy. HNO.

[9] Doty RL, Frye RE, Agrawal U (1989). Internal consistency reliability of the fractionated and whole University of Pennsylvania smell identification test. Percept Psychophys.

[10] Lötsch J, Hummel T (2019). Clinical usefulness of self-rated olfactory performance—a data science-based assessment of 6000 participants. Chem Senses.

[11] Weis S, Scherag A, Baier M (2021). Antibody response using six different serological assays in a completely PCR-tested community after a COVID-19 outbreak—the CoNAN study. Clin Microbiol.

[12] Hummel T, Kobal G, Gudziol H, Mackay-Sim A (2007). Normative data for the “Sniffin’ Sticks” including tests of odor identification, odor discrimination, and olfactory thresholds: an upgrade based on a group of more than 3,000 subjects. Eur Arch Otorhinolaryngol.

[13] Kobal G, Hummel T, Sekinger B, Barz S, Roscher S, Wolf S (1996). “Sniffin’ sticks”: screening of olfactory performance. Rhinology.

[14] Hummel T, Sekinger B, Wolf SR, Pauli E, Kobal G (1997). ‘Sniffin’ sticks’: olfactory performance assessed by the combined testing of odor identification, odor discrimination and olfactory threshold. Chem Senses.

[15] Oleszkiewicz A, Schriever VA, Croy I, Hahner A, Hummel T (2019). Updated Sniffin’ Sticks normative data based on an extended sample of 9139 subjects. Eur Arch Otorhinolaryngol.

[16] Gudziol H, Hummel T (2007). Normative values for the assessment of gustatory function using liquid tastants. Acta Otolaryngol.

[17] Tong JY, Wong A, Zhu D, Fastenberg JH, Tham T (2020). The prevalence of olfactory and gustatory dysfunction in COVID-19 participants: a systematic review and meta-analysis. Otolaryngol Head Neck Surg.

[18] Wehling E, Nordin S, Espeseth T, Reinvang I, Lundervold AJ (2011). Unawareness of olfactory dysfunction and its association with cognitive functioning in middle aged and old adults. Arch Clin Neuropsychol.

[19] Soter A, Kim J, Jackman A, Tourbier I, Kaul A, Doty RL (2008). Accuracy of self-report in detecting taste dysfunction. Laryngoscope.

[20] von Bartheld CS, Hagen MM, Butowt R (2020). Prevalence of chemosensory dysfunction in COVID-19 participants: a systematic review and meta-analysis reveals significant ethnic differences. ACS Chem Neurosci.

[21] Moein ST, Hashemian SM, Tabarsi P, Doty RL (2020). Prevalence and reversibility of smell dysfunction measured psychophysically in a cohort of COVID-19 participants. Int Forum Allergy Rhinol..

[22] Vaira LA, Hopkins C, Petrocelli M (2020). Smell and taste recovery in coronavirus disease 2019 participants: a 60-day objective and prospective study. J Laryngol Otol.

[23] Biadsee A, Dagan O, Ormianer Z, Kassem F, Masarwa S, Biadsee A (2021). Eight-month follow-up of olfactory and gustatory dysfunctions in recovered COVID-19 patients. Am J Otolaryngol.

[24] Nehme M, Braillard O, Chappuis F, Courvoisier DS, Guessous I (2021). Prevalence of symptoms more than seven months after diagnosis of symptomatic COVID-19 in an outpatient setting. Ann Intern Med.

[25] Renaud M, Thibault C, Le Normand F, Mcdonald EG, Gallix B, Debry C, Venkatasamy A (2021). Clinical outcomes for patients with anosmia 1 year after COVID-19 diagnosis. JAMA Netw Open.

[26] Lechien JR, Saussez S, Maniaci A, Vaira LA (2021). The study of recovery rates of COVID-19 olfactory and gustatory dysfunctions requires psychophysical evaluations. Am J Otolaryngol.

[27] Yang J, Pinto JM (2016). The epidemiology of olfactory disorders. Curr Otorhinolaryngol Rep.

[28] Neuland C, Bitter T, Marschner H, Gudziol H, Guntinas-Lichius O (2011). Health-related and specific olfaction-related quality of life in participants with chronic functional anosmia or severe hyposmia. Laryngoscope.

[29] Vaira LA, Lechien JR, Salzano G (2020). Gustatory dysfunction: a highly specific and smell-independent symptom of COVID-19. Indian J Otolaryngol Head Neck Surg.

[30] Lechien JR, Cabaraux P, Chiesa-Estomba CM (2020). Objective olfactory evaluation of self-reported loss of smell in a case series of 86 COVID-19 participants. Head Neck.

[31] Marschner H, Gudziol H, Guntinas-Lichius O (2020). Olfactory dysfunctions are substantially more frequent than they are complained. Laryngorhinootologie.

[32] Welge-Luessen A, Hummel T, Stojan T, Wolfensberger M (2005). What is the correlation between ratings and measures of olfactory function in participants with olfactory loss?. Am J Rhinol.

[33] Oleszkiewicz A, Hummel T (2019). Whose nose does not know? Demographical characterization of people unaware of anosmia. Eur Arch Otorhinolaryngol.

